# Identification of Faba bean genetic loci associated with quantitative resistance to the fungus *Botrytis fabae*, causal agent of chocolate spot

**DOI:** 10.3389/fpls.2024.1383396

**Published:** 2024-04-19

**Authors:** Anne Webb, Tom R. Reynolds, Tally I. C. Wright, Rosa Caiazzo, David C. Lloyd, Jane E. Thomas, Thomas A. Wood

**Affiliations:** ^1^ Plant Pathology, NIAB, Cambridge, United Kingdom; ^2^ Department of Biology, University of Oxford, Oxford, United Kingdom; ^3^ Technical Support, Illumina, Cambridge, United Kingdom; ^4^ Germinal Holdings, Institute of Biological, Environmental and Rural Sciences, Aberystwyth University, Aberystwyth, United Kingdom

**Keywords:** *Vicia faba*, chocolate spot, *Botrytis fabae*, quantitative trait locus, QTL

## Abstract

**Introduction:**

Chocolate spot, caused by the ascomycete fungus *Botrytis fabae*, is a devastating foliar disease and a major constraint on the quality and yield of faba beans (*Vicia faba*). The use of fungicides is the primary strategy for controlling the disease. However, high levels of partial genetic resistance have been identified and can be exploited to mitigate the disease.

**Methods:**

The partially resistant *V. faba* cultivar Maris Bead and susceptible Egyptian accession ig70726 were crossed, and a genetic mapping population of 184 individuals was genotyped in the F_2_ generation and screened for resistance to *B. fabae* infection in the F_3_, F_5_, and F_6_ generations in a series of field experiments. A high-density linkage map of *V. faba* containing 3897 DArT markers spanning 1713.7 cM was constructed.

**Results:**

Multiple candidate quantitative trait loci (QTLs) in 11 separate regions of the *V. faba* genome were identified; some on chromosomes 2, 3, and 6 overlapped with loci previously linked to resistance to Ascochyta leaf and pod blight caused by the necrotrophic fungus *Ascochyta fabae*. A transcriptomics experiment was conducted at 18 h post-inoculation in seedlings of both parents of the mapping population, identifying several differentially expressed transcripts potentially involved in early stage defence against *B. fabae*, including cell-wall associated protein kinases, NLR genes, and genes involved in metabolism and response to reactive oxygen species.

**Discussion:**

This study identified several novel candidate QTLs in the *V. faba* genome that contribute to partial resistance to chocolate spot, but differences between growing seasons highlighted the importance of multi-year phenotyping experiments when searching for candidate QTLs for partial resistance.

## 1 Introduction

Faba bean (*Vicia faba* L.) is grown as a pulse crop for human consumption and animal feed in temperate and subtropical regions worldwide with an estimated 2.67 m ha under production in 2020 for dry broad bean and horse bean (FAOSTAT, 2022). In the United Kingdom, approximately 190,000 ha of field beans are cultivated annually, generating 700,000 t of seed (4.2 t/ha average yield). *V. faba* contributes to soil fertility by improving soil structure and providing residual nitrogen to subsequent crops in a rotation due to its ability to fix atmospheric nitrogen.

Faba bean production is negatively affected by various foliar pathogens including *Ascochyta fabae* (Ascochyta blight), *Peronospora viciae* (bean downy mildew), *Cercospora zonata* (leaf spot), and chocolate spot, caused by *Botrytis fabae* Sard. Chocolate spot is one of the most important and potentially yield-limiting foliar diseases of faba bean, and is known to cause severe yield losses in major bean-producing areas worldwide (Stoddard et al., 2010; Abo-Hegazy et al., 2012; Wakoya and Abdissa, 2022). New chocolate spot disease outbreaks are initiated by conidia, from infected plant debris or seeds, where robust sclerotia can persist for many months or even years (Harrison, 1979, 1981). Conidia are disseminated by wind and rain-splash under high humidity, rainfall, extended periods of leaf wetness, and wind (Harrison, 1980; Harrison, 1983; Harrison, 1984; Fitt et al., 1985). Chocolate spot disease progresses in two stages with small, discrete lesions appearing on leaves first during the “non-aggressive stage” (Deverall and Wood, 1961). Under continuing optimal conditions, the disease progresses rapidly to the “aggressive stage.” Small lesions multiply and spread to younger parts of the plant, followed by the appearance of large necrotic and sporulating patches on older foliage, leading to progressive defoliation beginning in the oldest parts of the plant.

Chocolate spots are primarily controlled by repeated preventive fungicide application. Established disease has been found to be difficult to control, with yield losses of 25% (approximately 1 t per hectare), and as high as 50% in untreated crops in the UK (Ward, 2016). Integrated Pest Management (IPM), crop rotation selection of healthy seeds, and intercropping have been found to slow the development of chocolate spots alongside frequent fungicide applications (Sahile et al., 2008; Stoddard et al., 2010).

Breeding for greater chocolate spot resistance has been confounded by complex genetic × environment interactions within field disease assessments (Hanounik and Robertson, 1988; Bouhassan et al., 2004; Villegas-Fernández et al., 2009, 2012). Multiple studies have found partial resistance across genotypes from diverse geographic origins (Elliott and Whittington, 1979; Hanounik and Robertson, 1988; Bond et al., 1993; Bouhassan et al., 2004; Villegas-Fernández et al., 2009; Beyene et al., 2018), sometimes confined to only some of the environments in which accessions were trialled (Hanounik and Robertson, 1988). These findings suggest that resistance to chocolate spots is multigenic and quantitative (Elliott and Whittington, 1979). Most contemporary commercial faba bean varieties exhibit limited resistance to chocolate spots (PGRO, 2021).

The faba bean is a diploid species with a large (13 Gb, n = 6) genome containing extensive regions of repetitive DNA (Jayakodi et al., 2023). Next-generation sequencing of *V. faba* transcriptomes has allowed large-scale mining of potential markers, facilitating the development of Simple-Sequence-Repeat (SSR) (El-Rodeny et al., 2014), single nucleotide polymorphism (SNP)-based Kompetitive Allele Specific PCR (KASP) markers (Cottage et al., 2012; Webb et al., 2016), high-density Axiom SNP genotyping arrays (Khazaei et al., 2021), and Specific Primer Enrichment Technology (SPET; Barchi et al., 2019) for faba bean. Consequently, many high-density linkage maps have been published, allowing the identification of quantitative trait loci (QTLs) for multiple disease resistance-related traits, e.g., bean rust (Ijaz et al., 2018), ascochyta blight, broomrape (Gutierrez and Torres, 2021), and chocolate spot (Gela et al., 2022). In a controlled environment inoculation experiment, Gela et al. (2022) identified five QTLs linked to resistance to chocolate spots on chromosomes 1 and 6 in an advanced recombinant inbred line (RIL) population derived from a cross between susceptible French cv. Mélodie and partially resistant Colombian accession ILB938.

In the UK, the tic bean cv. Maris Bead has long been known to be partially resistant to chocolate spot in the field, producing numerous small lesions, with aggressive lesions being “uncommon” (Harrison, 1979, 1980, 1981). This characteristic enables plants to survive longer periods of weather that are conducive to chocolate spot than fully susceptible varieties. In this study we focussed on identifying QTLs linked to partial resistance to chocolate spot in a bi-parental F_2:3:5:6_ population derived from a cross between the partially resistant inbred line NV640, derived from British cv. Maris Bead and susceptible inbred line NV293, derived from susceptible Egyptian accession ig70726. Three generations (F_3_, F_5,_ and F_6_) were tested under field conditions in three independent seasons. In addition, the early responses of faba beans to *B. fabae* between NV640 and NV293 were evaluated in a differential expression experiment and compared to the results of QTL mapping.

## 2 Materials and methods

### 2.1 Plant materials and development of mapping population

The parental lines were inbred for a minimum of eight generations to ensure high levels of homozygosity. The susceptible *V. faba* inbred line NV293, derived from the Egyptian accession ig70726 (ICARDA), was crossed with NV640, an inbred partially resistant line derived from the UK cultivar Maris Bead. Of the 361 individuals in the F_2_ generation, 184 produced sufficient F_3_ seeds to be included in the field experiments. These were genotyped and advanced as inbred lines for phenotyping in the F_3_, F_5,_ and F_6_ generations, with one individual from each F_2_ individual taken forward to the next generation.

### 2.2 Genotyping and map construction

DNA was extracted from 184 F_2_ individuals from the mapping population and the two parental lines. Young leaflets were collected from F_2_ seedlings, frozen at –80°C and homogenised in a 1600 MiniG tissue homogeniser (SPEX SamplePrep, Stanmore) before DNA extraction, following the protocol of Doyle and Doyle (1987), with 2% v/v RNAse A (7,000 u/ml, QIAGEN) added to the extraction buffer immediately prior to extraction.

The F_2_ plants and parental lines were genotyped using DArT sequencing (Wenzl et al., 2004) at medium density according to the manufacturer’s specifications, yielding 35,585 genetic markers. Only the co-dominant SNPs were used for mapping. Parent genotypes were replicated twice and genetic markers between replicates that were missing or inconsistent were discarded, leaving 33,873 markers. Markers were also removed if they were: monomorphic, had technical replicate reproducibility (repAvg, assessed by DArT as part of marker-QC) <0.95, and had >10% missing values. After the initial quality control, 3,938 markers remained.

Markers showing excessive segregation distortion (>0.05) were removed from the genetic map construction (Taylor and Butler, 2017). Genotypes with excessive genetic crossovers and >10% missing calls were also excluded. The linkage map was constructed using the R/ASMap v. 1.0.4 (Taylor and Butler, 2017), and R/qtl v. 1.5 (Broman et al., 2003) in R v.3.4 (Anonymous, 2017). Linkage groups were ordered using the function ‘formLinkageGroups’ with max.rf set to 0.35, minimum logarithm-of-odds score (LOD) set to 19 and using the map function ‘Kosambi.’ Linkage groups were refined using the ASMap function ‘mstmap.cross’ using default parameters, followed by ‘droponemarker’ to remove markers with weak linkage. This placed 1,111, 648, 634, 568, 491, and 460 markers in the six linkage groups, leaving 26 unmapped markers that were excluded from subsequent analyses. During final reordering, 33 genotypes and 15 markers were removed. The linkage groups were named according to the corresponding *V. faba* chromosomes (**Supplementary Table S1**). Linkage maps were drawn using the R-package LinkageMapView 2.1.2 (Ouellette et al., 2018).

### 2.3 Genetic map synteny to other legume species and linkage maps

To anchor the map to *V. faba*, *Medicago truncatula*, and chickpea, marker sequences were searched against the complete coding sequences (CDS) of the *V. faba* assembly for cultivar Hedin GCA_948472305.1 (Jayakodi et al., 2023), using BLASTn v.2.2.29 (Altschul et al., 1990; Camacho et al., 2009) and CDS of *M truncatula* A17 assembly v. 4.0 (Young et al., 2011), with parameter -task blastn-short, e-value set to 1e−5, and parameter -num-alignments set to 2. QTLs from the linkage map of Gela et al. (2022) were anchored according to their marker coordinates within the *V. faba* genome. In addition, all mapped markers were searched on the Legume Information system’s blastn-server (Gonzales et al., 2005) running BLASTn with default settings against the CDS databases of *M. truncatula* A17v5 (Pecrix et al., 2018; medtr.A17.gnm5.ann1_6.L2RX) and *Cicer arietinum* Frontier v1 (Varshney et al., 2013; cicar.CDCFrontier.gnm1.GkHc).

### 2.4 Functional gene annotations

Predicted proteins of *V. faba* were functionally annotated using the standalone version of InterProScan-5.59-91.0 (Jones et al., 2014) with all databases enabled.

### 2.5 Collection, cultivation, and inoculation of plants with *Botrytis fabae*

**Figure 1:**
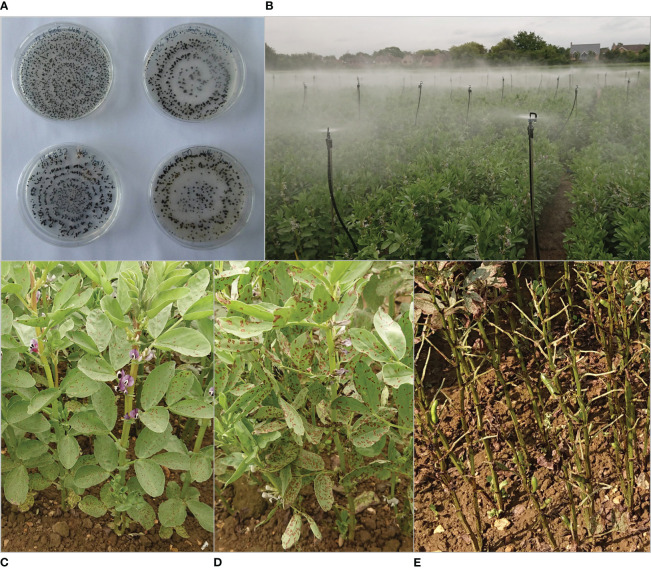
*Botrytis fabae* forming sclerotia on potato dextrose agar **(A)**, field experiment misted to ensure high ambient humidity **(B)**, non-aggressive stage of chocolate spot on partially resistant line NV640 of *Vicia faba* **(C)**, transition to aggressive stage on susceptible line **(D)**, extensive defoliation and early senescence of host plants, and late stage of the disease **(E)**.

Isolates of *B. fabae* were collected from farm-saved *V. faba* seed samples. Surface-sterilised seeds were plated on potato dextrose agar (PDA) and incubated at 20°C for five to seven days (**Figure 1A**). Colonies of *Botrytis* spp. were transferred to new plates and identified as *B. cinerea* or *B. fabae* by the size and shape of conidia as described in Harrison and Heilbronn (1988), with *B. fabae* having slightly larger and ovoid conidia measuring between 15 μm and 25 µm while *B. cinerea* produced round conidia of 7.5 µm to 12.5 µm diameter (Waller and Ellis, 1974a, b). Single-spore isolates were obtained from each culture and stored in microcentrifuge tubes as dried sclerotia at 4°C. An equal quantity of 10 isolates (Bf-126678-1, Bf-125816 A, Bf-123698 A, Bf-124342, Bf-124967 A, Bf-125390A, Bf-124305-1, Bf-124137-1, Bf-126384, Bf-124662) were used for all inoculations. *B. fabae* cultures were initially isolated from infected, UK-grown faba bean seeds.

Sclerotia were plated on PDA, and emerging cultures were transferred to modified sporulation medium (Leach and Moore, 1966). The medium was prepared by reducing the amount of sucrose to 100 g/L and incubating under UV light at 20°C for at least 10 days. Approximately 5 ml distilled water was added to each plate, and spores were gently scraped off from the colony surface into a suspension. 1% (w/v) of sucrose was added to the spore suspension prior to inoculation.

Plants were sprayed with 4 ml spore suspension per plant at a concentration of at least 1 × 10^5^ spores/mL. Field trials were inoculated twice within one week, when possible, during periods of rainfall, with the first inoculation at early flowering in 2017, early podding in 2019, and at the four- to eight-true-leaf stage in 2021. Trials were misted for 2 min from the time of the first inoculation at three-hour intervals during nighttime to increase humidity.

### 2.6 Disease assessments

Field experiments were conducted over three years with the F_3_ RILs in 2017, F_5_ RILs in 2019, and F_6_ RILs in 2021. As each RIL generation advanced, the seeds were always harvested from a single plant. Experiments were conducted with at least 150 RILs and controls randomised over three replicates, with incomplete blocks of 16 plots nested within replicates. The plots consisted of a row of five plants planted 10 cm apart. Four plots were combined into one 2 m long row bounded by two discarded plants at either end. Each block consisted of eight rows with 0.5 m wide paths between every four rows. Misters were installed 1.5 m apart in 1 m wide paths perpendicular to each replicate and on the outer edges of the experiment (**Figure 1B**). Chocolate spot symptoms were scored for each replicate plot as the percentage of necrotic leaf area and defoliation at later disease stages (**Figures 1C–E**). The scoring intervals varied depending on the progress of symptoms, with intervals between one and 15 days. Disease was scored four to 10 times to effectively capture the entire development of the disease throughout the seasons. Areas under the disease progression curve (AUDPCs) were calculated from all recorded scores (Vanderplank, 1963). Daily readings for total precipitation, average relative humidity, wind speed, and average temperature were obtained for the trial periods from a weather station located at NIAB, Cambridge, UK (543500E 260600N, Lat 52.245 Lon 0.102, 26m above sea level).

### 2.7 QTL-mapping and enrichment analysis

Best linear unbiased predictors (BLUPs) for disease symptoms were calculated using the R package SpATS (Rodríguez-Álvarez et al., 2018) for each time point and annual AUDPCs using a spatial model including the blocking factors ‘rep’ and ‘block.’ Genotype was treated as a random factor to obtain BLUPs using the ‘predict.SpATS’ function. Generalised heritability was estimated using the ‘getHeritability’ function (Oakey et al., 2006). QTLs were mapped in R v. 4.1.0 using functions within the R package qtl v. 1.5 (Broman et al., 2003) against the NV640 × NV293 linkage map. Single candidate loci were identified using the ‘scanone’ function with Haley–Knott regression. Thresholds were calculated using 5,000 permutations for alpha at 0.01, 0.05, and 0.1. All QTLs above alpha 0.1 were refined using the functions ‘makeqtl’ and ‘refineqtl.’ Additional QTLs were searched using the function ‘addqtl’ followed by ‘addint’ to explore interactions where multiple candidate QTLs were detected. QTLs exceeding the 0.05 threshold calculated for scanone were retained in the final model and percentage phenotypic variation explained for each QTL was estimated using ‘fitqtl.’ Flanking markers were detected with ‘lodint,’ using a 1.5 LOD drop. For each of the three field trials, QTLs were reported for individual scores and AUDPCs. QTL peak and interval genetic map locations were anchored to genomic locations on the *V. faba* genome (Jayakodi et al., 2023) using markers mapped to *V. faba* coding sequences, providing estimates of the number of genes within each QTL interval. Gene ontology (GO) terms for genes underlying QTL clusters with overlapping flanking regions were tested for enrichment using AgriGO v2.0 (Du et al., 2010; Tian et al., 2017). Due to the low number of seeds obtained from each plant, phenotyping had to be conducted using seeds sourced from three RIL generations. As the number of heterozygous genotypes halves at each generation of inbreeding, the true calls at F6 could not be determined without genotyping data from a later generation. To account for this effect, all QTL-scans for the F_6_ data were recalculated with a heterozygous F_2_-calls set to missing to determine if the identified loci were recovered.

### 2.8 Differential gene expression

Seeds of lines NV293 and NV640 were planted into trays with Levington M2 compost and maintained in a growth room with 12 h light at 19°C/11°C. Seedlings were inoculated at the four-true leaf stage with a spore suspension of *B. fabae* isolate Bf 124967 at a concentration of 2 × 10^4^ spores/mL with 1% (w/v) sucrose and then sprayed with distilled water. Trays were covered with clear polythene after inoculation until sampling. Approximately 50 mg–100 mg of young leaflets was sampled from three biological replicates at 33 h post-inoculation (hpi) when both partially resistant and susceptible inoculated seedlings showed clear (but contrasting) chocolate spot symptoms, and were frozen in liquid nitrogen. RNA was extracted using the RNeasy Mini Plant kit (Qiagen), following the manufacturer’s instructions, including DNase I on-column digestion. Total mRNA was sequenced as 150 bp paired-end reads on an Illumina HiSeq 2500 platform and trimmed for adapters and quality (phred <30) at the Centre for Genomic Research, University of Liverpool. Trimmed reads were submitted to the European Nucleotide Archive (ENA) under the project accession number PRJEB58257. Reads were counted against the reference genome GCA_948472305.1, using rsem-calculate-expression (RSEM v. 1.2.28), using hisat2 v. 2.2.1 as aligner (Li and Dewey, 2011; Kim et al., 2015). Differential expression (i) between varieties regardless of treatment and (ii) varieties in response to infection at 33 hpi was analysed using the R package DESeq2 v. 1.38.3 (Love et al., 2014). Genes were considered differentially expressed if the adjusted p-value (Benjamini and Hochberg, 1995) was smaller than 0.05. Log-fold changes were shrunk for plotting using the packages apeglm v. 1.20.0 (Zhu et al., 2019), and ashr v. 2.2-63 (Stephens, 2017). Heatmaps were generated using package Pheatmap v. 1.0.12.

## 3 Results

### 3.1 Disease assessments

Chocolate spot symptoms developed at different speeds over the years, with final scores obtained 26, 13, and 73 days after inoculation in 2017, 2019, and 2021, respectively. Disease progress slowed in dry and sunny weather and accelerated after periods of rain as a natural response to conditions that were not conducive to disease, as anticipated (**Supplementary Figure S1**). In susceptible lines, disease progression typically starts with a few discrete chocolate-coloured spots appearing on green leaves. Later, lesions multiplied quickly, with additional large, grey necrotic patches appearing on older leaves first, leading to defoliation progressing upward from the oldest leaves, leading to premature death (**Figures 1C, D**).

The partially resistant parental line NV640 showed the slowest disease progression throughout the trials, resulting in the lowest AUDPC, whereas the chocolate spot progressed rapidly in line NV293, with its AUDPC being the highest each year (**Supplementary Table S7**). The ranking of progeny lines varied somewhat between years, reflected in the AUDPC correlation values of between 0.17 and 0.34 (**Supplementary Figure 2**). No progeny line consistently showed higher or lower scores or AUDPCs than either parent, remaining within the range defined by NV640 and NV293. Generalised heritability was estimated to range from 0.36 to 0.85, for the scoring dates at which QTLs could be mapped (**Table 1**).

**Table 1:** QTLs for BLUPs of individual scores and area under the disease curve (AUDPC, shown in bold) identified in the NV640 × NV293 population, field experiments 2017 (F_3_), 2019 (F_5_), and 2021 (F_6_).

Year	Foliar disease, score	% leaf coverageNV293 (S)	% leaf coverageNV640 (R)	Developmentalstage	QTL name	LOD	QTL interval (cM start, cM end)	Trait heritability (H^2^)	% variation explained	*V. faba* genes
2017	1–11 d.a.i.	0.9–4.0	1.0–4.0	podding	none			0.06–0.21		
	13 d.a.i.	39.0	23.4	podding	LG3@119.2 LG5@107.3 LG6@169.6	4.983 5.672 3.804	72.8, 136.5 92.9, 115.7 141.6, 181.0	0.36	26.6	1,468 414 642
	15 d.a.i.	50.4	29.1	podding	LG3@75.2aLG5@86.9	5.276 6.535	70.5, 121.2 78.0, 95.3	0.49	22.9	1,214 581
	19 d.a.i.	80.2	52.5	podding	LG2@68.4 LG3@115.2 LG4@135.3 LG5@93.9	4.334 6.709 4.675 10.500	66.1, 70.1 109.5, 118.5 132.9, 136.3 76.3, 95.3	0.50	40.8	204 293 106 645
	21–26 d.a.i.	90.8–98.1	82.9–97.1	podding	none			0.25–0.34		
	**AUDPC**	**1117**	**715**	**podding**	**LG3@75.2bLG5@78.6**	**4.740** **8.527**	**70.5, 139.8** **74.3, 96.2**	**0.55**	**25.9**	**1,496** **756**
2019	3 d.a.i.	7.2	3.6	flowering	LG3@144.5	4.337	132.5, 157.5	0.41	10.6	437
	7 d.a.i.	88.4	60.5	flowering	LG2@50.1 LG3@136.5	5.557 4.988	42.7, 52.1 104.8, 153.2	0.41	21.9	121 380
	10 d.a.i.	95.7	81.0	podding	LG2@102.1	5.084	74.8, 171.4	0.47	12.4	2,077
	13 d.a.i.	96.9	88.3	podding	LG2@10.7 LG2@171.4	4.137 5.505	0.0, 50.1 164.1, 173.1	0.39	21.1	781 301
	**AUDPC**	**751**	**684**	**podding**	**LG2@169.8**	**5.317**	**152.8, 173.1**	**0.41**	**12.9**	**449**
2021	3–8 d.a.i.	0.6–3.3	0.6–2.7	vegetative	none			0.10–0.19		
	15 d.a.i.	7.0	2.9	vegetative	LG1@168.9 LG3@27.7	3.089 4.355	42.0, 195.6 13.6, 36.0	0.77	22.9	2,478 250
	22 d.a.i.	11.8	2.6	flowering	none			0.82		
	32 d.a.i.	30.9	6.7	flowering	LG2@84.5 LG6@121.0	3.675 5.331	55.4, 90.5 119.3, 149.9	0.81	25.0	695 553
	44 d.a.i.	75.0	10.4	podding	LG3@24.7 LG6@110.0a	5.119 5.801	20.3, 37.7 106.3, 117.6	0.85	24.7	409 207
	59–73 d.a.i.	98.9–99.8	27.0–52.5	podding	none			0.75–0.80		
	**AUDPC**	**3,602**	**896**	**podding**	**LG3@28.4** **LG6@110.0b**	**5.568** **5.504**	**20.3, 37.7** **105.6, 117.0**	**0.89**	**26.1**	**196** **409**

Lower-case letters added to QTL names distinguish multiple QTLs with identical peak loci. Resistance was associated with NV640 allele for all QTLs except LG1@168.9 at 15 d.a.i., 2021. S, susceptible parent; R, resistant parent; AUDPC, areas under the disease progress curve; d.a.i., days after inoculation; LOD, logarithm of odds.

### 3.2 Linkage map and synteny

The linkage map for NV640 × NV293 consisted of six linkage groups with a total of 3,897 genetic markers and a total length of 1,713.7 cM with no gaps exceeding 6.6 cM in length and average marker spacing of 0.4 cM. The first published genome assembly of the *V. faba* cv. Hedin (Jayakodi et al., 2023) allows linkage groups to be anchored to the corresponding chromosomes and genes in the *V. faba* genome assembly. The linkage map was anchored to the *V. faba* genome assembly with 1,515 markers located on one or more annotated genes (**Supplementary Table S1**). Marker sequences and the NV640 × NV293 linkage map are available under https://www.viciatoolbox.org.

### 3.3 QTLs and associated genes

**Figure 2:**
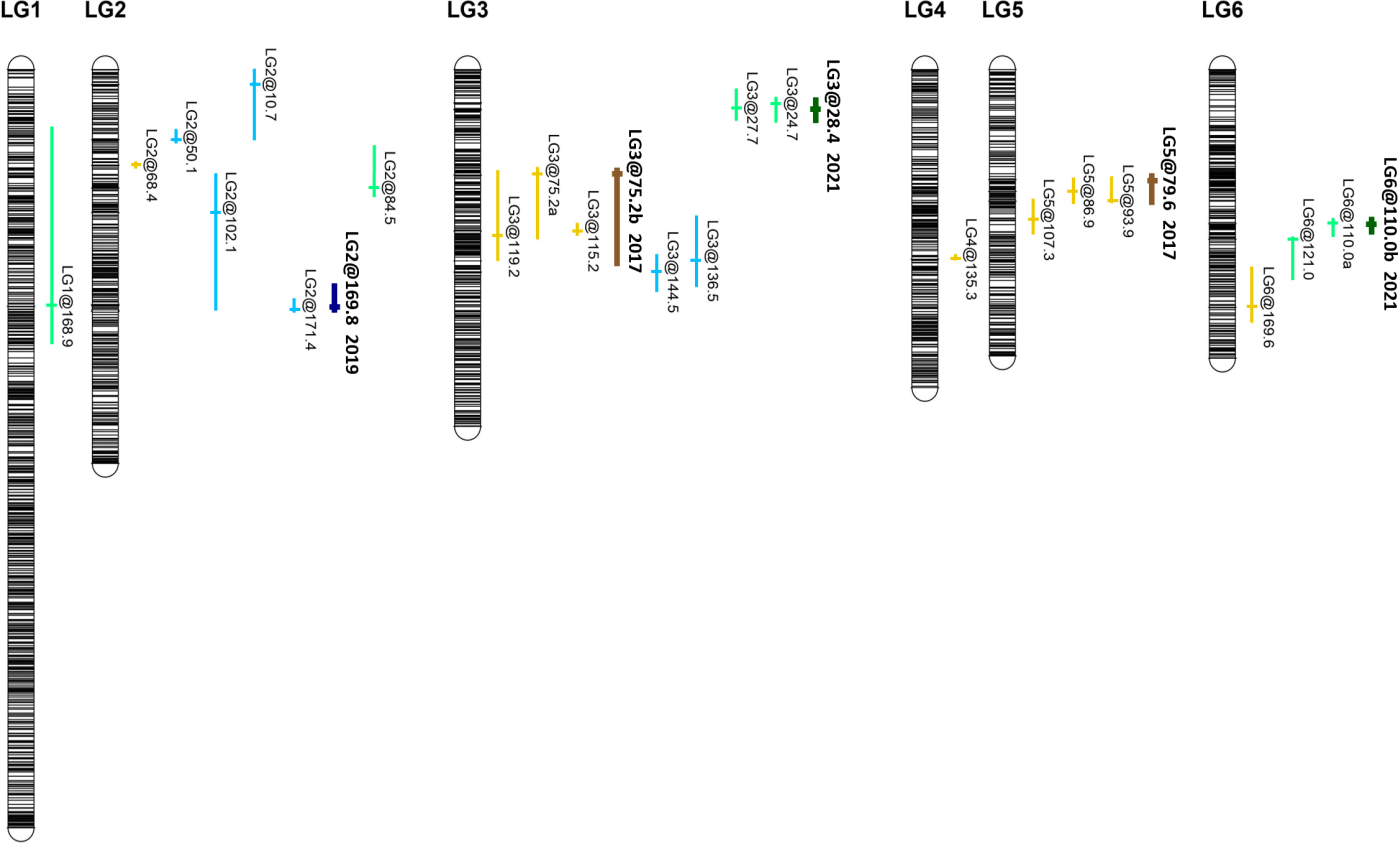
Genetic map locations of QTL identified for chocolate spot resistance on the linkage map of *V. faba* NV640 × NV293 in trials conducted in 2017, 2019, and 2021. QTL associated with individual scores are shown in light shades, representing individual scores, and dark shades represent QTL associated with areas under the disease progress curve (AUDPCs). Colours represent years: 2017 brown, 2019 blue, and 2021 green.

Multiple putative QTLs were identified for the AUDPCs and at individual disease scoring points (**Figure 2**). In 2017, two QTLs on LG3 (at 75.2 cM) and LG5 (78.6 cM) collectively accounted for 25.9% of the observed variation in AUDPCs. In 2019, one QTL was found on LG2 at 169.8 cM, accounting for 12.9% of the variation, and two in 2021 on LG3 at 28.4 cM and one on LG6 at 110.0 cM estimated to collectively represent 26.1% of the observed variation. QTLs for individual disease scores were found on LG2 and LG3 with intervals on LG3 partly overlapping with QTLs identified in 2017. The intervals were relatively broad, encompassing 196–1,496 *V. faba* genes (**Table 1**, **Supplementary Table S2**). When comparing individual phenotypic scores, different QTLs were identified at different stages of the disease and between trial years; seven of these did not overlap with any of the AUDPC QTLs, including those on LG1 and LG4 and a cluster on LG2 (**Table 1**). Significant QTLs were identified during the periods when symptoms progressed rapidly in the susceptible parent, while progression in the partially resistant parent was delayed; no QTLs were observed during very early disease development, except in 2019, and during the late stages of the disease. Symptom severity in the resistant parent NV640 and the entire progeny eventually reached that of the susceptible NV293 parent as conditions favourable to *B. fabae* were maintained throughout the experiment (**Supplementary Figure S1**). QTL intervals were broad with multiple peaks crossing the significance threshold, especially for the QTLs on LGs 2, 3, and 6 (**Supplementary Figures S3**, **S4**), with estimated QTL-peak locations shifting between scores within and between years, indicating that multiple independent genes could contribute to enhanced resistance in NV640. No significant interaction effects were identified for any QTLs, therefore, the effects of all QTLs were considered to be additive (**Supplementary Table S2**). With the exception of QTL LG1@168.9, which was identified at 15 d.a.i. in 2017, resistance to *B. fabae* appeared to be conferred by NV640 (**Supplementary Figures S5**-**S7**).

QTL intervals on LGs 1, 2, 3, and 5 covered several large clusters of NLRs, leucine-rich repeat-containing receptor-like protein kinases, including many wall-associated receptor kinases (WAKs) interspersed with chitin-receptors (**Table 2**), and clusters of *GNK2*-class genes associated with antifungal/salt stress responses. In Linkage Group 2, the regions covered by overlapping QTLs LG2@68.4 and LG2@84.5 were enriched for processes related to transcription and regulation, and QTLs LG2@10.7, LG3@75.2ab, and cluster LG3@115.2/LG3@119.2 were enriched for GO terms associated with defence. QTLs LG1@168.9, LG2@10.7, LG2@84.5, LG2@169.8, LG2@171.4, LG3@75.2ab, LG3@115.2, LG3@119.2, and all QTLs on LG5 are enricher for terms relating to vesicular or transmembrane transport (**Supplementary Table S4**). Chitin receptors were clustered under the QTLs LG1@168.9, LG2@68.4, LG2@84.5, and LG121.1 (**Table 2**).

**Table 2:** Putative pathogen response candidate genes located within QTL intervals on chromosomes of *V. faba*.

Chromosome	QTLs	Class	Candidates	Total	Examples
Chr1	LG1@168.9	NLR/disease resistance genes	41	93	TNLs, CNLs
		wall-associated kinases	6	18	cluster of wall-associated galacturonan-binding receptor kinases, annotated as *Lr10* and OS01G0690200 protein
		chitin receptors	4	18	LysM domain receptor kinase *CERK1*/*LYK3*-like, clustered with disease resistance genes
		antifungal/salt stress	5	13	cluster of promastigote surface antigen proteins PSA *Gnk2*-like
Chr2	LG2@10.7	disease resistance genes	46	95	clusters of CNLs, TNLs
		antifungal/salt stress	7	13	cluster of 6 promastigote surface antigen proteins PSA
		cytochrome P450	20	52	cytochrome P450 98A9, 26, 71B11-related
Chr2	LG2@68.4, LG2@84.5	disease resistance genes	6	95	*EDS1* gene, one RNL,
		antifungal/salt stress	6	13	cluster of cysteine-rich receptor-like protein kinases 3
		chitin receptors	2	4	LysM domain receptor kinase *CERK1*/*LYK3*-like
		cytochrome P450	6	52	cytochrome P450 26, 703A2-related-related
Chr2	LG2@102.1	disease resistance genes	21	95	TNLs, 2 *EDS1* genes
		wall-associated kinases	5	7	wall-associated receptor kinases galacturonan-binding 21
		chitin receptors	1	4	LysM domain receptor kinase *CERK1*/*LYK3*-like
Chr3	LG3@24.7, LG3@27.7, LG3@28.4	Chitinases	2	10	under peak LG3@27.7, Glycosyl hydrolases family 18, chitinase 2-like
		cytochrome P450	12	46	cytochrome P450 76C, polypeptide 5-related, flavonoid 3’-monooxygenase-related
Chr3	LG3@75.2, LG3@115.2, LG3@119.2, LG3@136.5	disease resistance genes	8	49	TNLs, protein enhanced disease resistance 2-like, 2 protein negative regulators of resistance, *NPR1*-interacting
		Chitinases	6	10	cluster of glycosyl hydrolases family 18
		xylanase inhibitors	7	11	Aspartyl proteases and aspartyl protease-like
		Barwin-like endoglucanases	7	9	cluster of pathogenesis-related protein-4, *WIN-2*
		legume lectins	5	8	cluster of concanavalin A-like lectins/glucanases
		cytochrome P450	6	46	cytochrome P450 76C, polypeptide 5-related
Chr3	LG3@136.5, LG3@144.5	antifungal/salt stress	13	16	large cluster of promastigote surface antigen proteins PSA, cysteine rich repeat secretory proteins, receptor-like serine/threonine protein kinases *SD1-8*
Chr4	LG4@135.3	cytochrome P450	8	38	cluster of 8 cytochrome P450 71 or 71B11-related in peak region
Chr5	LG5@79.6, LG5@86.9, LG5@93.9	disease resistance genes	5	29	cluster of wall-associated galacturonan-binding receptor kinases, incl. 2 *Lr10*
		WRKY-transcription factors	6	14	under peak LG5@79.6 *WRKY1*-related; *WRKY30*, *WRKY21*
		Barwin-like endoglucanases	10	19	
Chr6	LG6@110.0a and b	cytochrome P450	5	13	Cytochrome P450 26, 93A3-like
Chr6	LG6@121.0, LG6@169.6	cytochrome P450	8	13	Cytochrome P450 26, 93A3-like and flavonoid 3’-monooxygenase-related
Chr6	LG6@169.6	antifungal/salt stress	1	2	Prohibitin
		WRKY-transcription factors	5	11	*WRK1*-related, *WRKY30*-related

### 3.4 Differential expression

**Figure 3:**
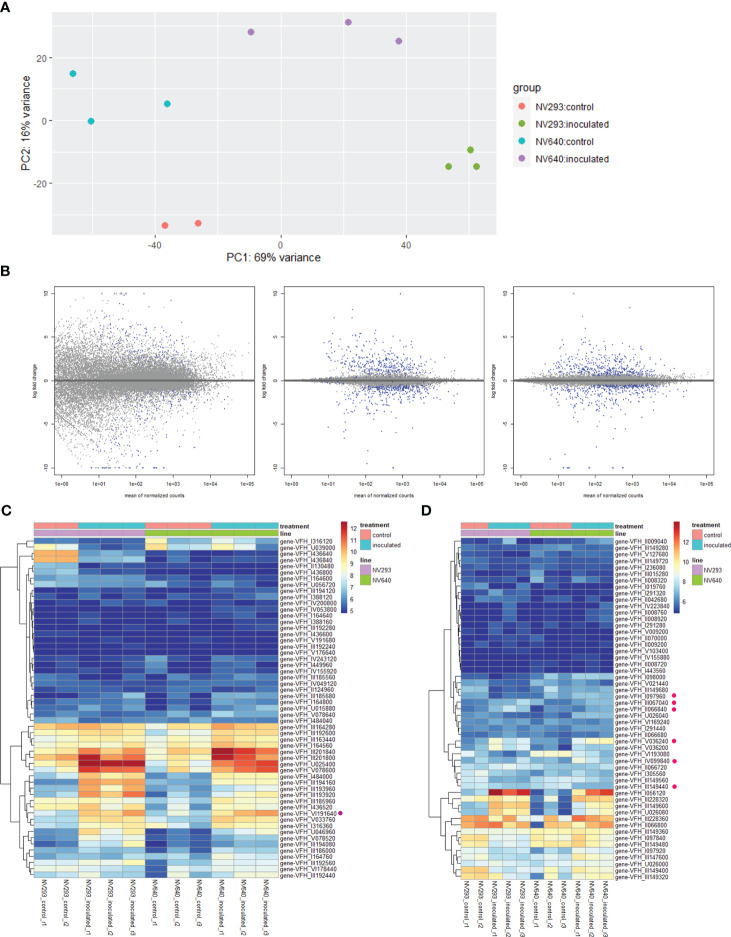
**(A)** PCA of sample replicates, DESeq2, 33 hpi; **(B)** log-fold change (lfc) plots, differentially expressed transcripts between NV293 and NV640 in response to inoculation with *B. fabae*: raw (left), normalised with *apeglm* (centre), and *ashr* (right). Blue dots indicate significant differential expression (adjusted p <0.1). **(C)** Heatmap of normalised counts (*vsd*) of expressed wall-associated *LR10*-like kinases. Purple dot: first-ranked differentially expressed gene-VFH_III_194160 (Vfaba.Hedin2.R1.3g194160.1), **(D)** heatmap of normalised counts (*vsd*) of expressed salt stress–response/antifungal genes. Pink dots: differentially expressed between NV640 and NV293 in response to *B*. *fabae*.

**Figure 4:**
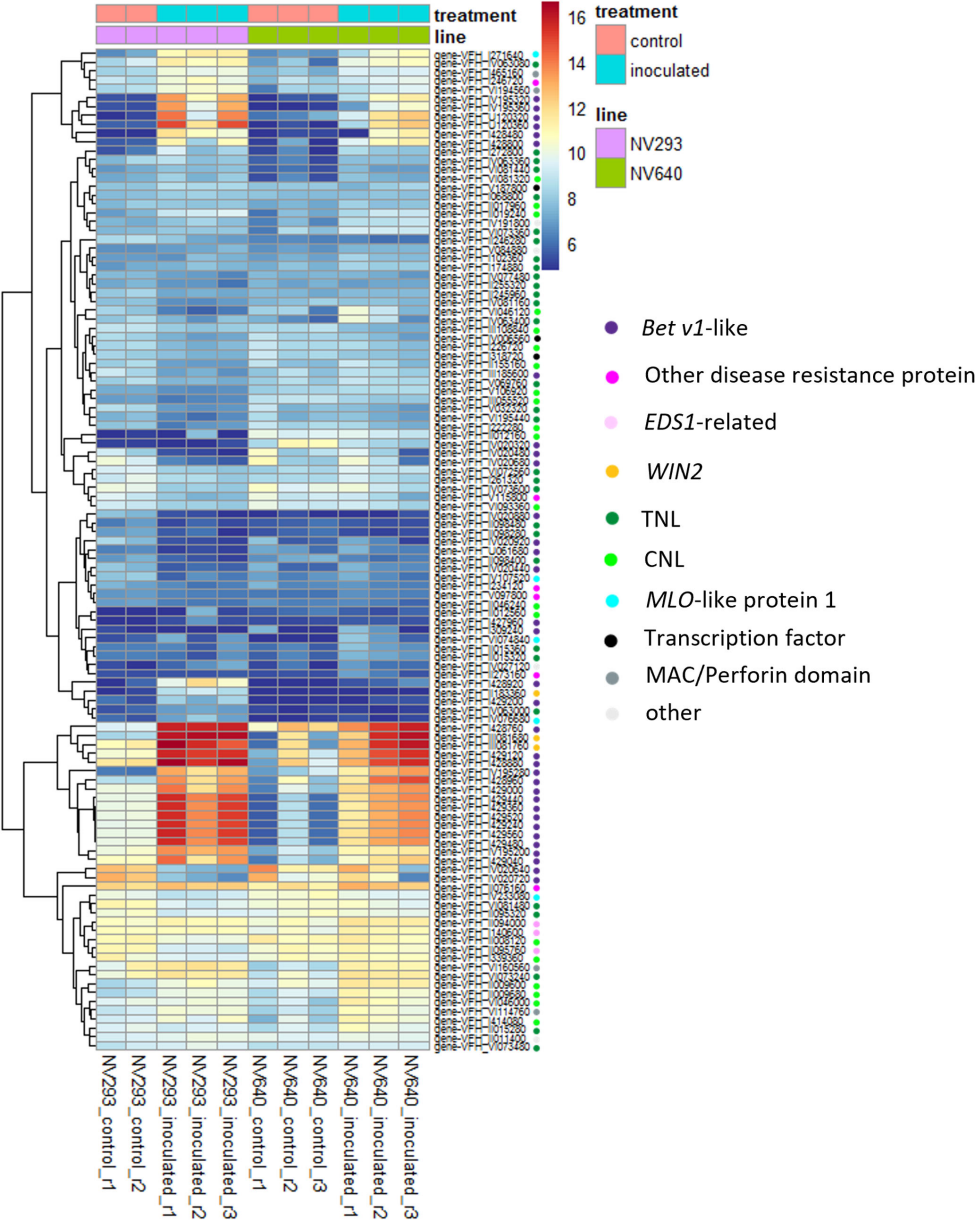
Heatmap of normalised counts (*vsd*) of expressed genes associated with GO:0006952 (defence response), differentially expressed between control and inoculated samples in at least one line. Coloured dots indicate functional annotations.

Differential expression analysis was conducted at an early stage of disease development at 33 h post-inoculation (hpi), at the appearance of the first symptoms in seedlings of both lines at the four-leaf stage. The reads were counted against the genes of *V. faba* cv. Hedin2 (**Figures 3A–D**). A total of 535 transcripts showed differential responses between NV293 and NV640 in response to infection with *B. fabae* (**Supplementary Table S5**). Of the differentially expressed transcripts, 155 were covered by QTLs, with all significant intervals represented. The most significantly differentially expressed gene Vfaba.Hedin2.R1.3g194160.1 encodes a wall-associated galacturonan-binding *Lr10*-like kinase (WAK), which is upregulated in both NV293 and NV640, with the response being much stronger in NV293 and is the only differentially expressed WAK (**Tables S5**-**S7**). A total of 28 of 55 WAKs were significantly up- or downregulated in the inoculated treatments in at least one of the lines, with most of them upregulated in response to *B. fabae* (**Supplementary Tables S5**-**S7**, **Figure 3C**). Of the 58 transcripts annotated for “Salt stress response/antifungal” functions, six showed a positive log-fold change for NV640 compared to NV293 (**Supplementary Table S5**, **Figure 3D**), five of which were located under QTLs LG1@168.9, LG3@136.5, and LG3@144.5. All of these genes were upregulated or unchanged in NV640 and downregulated in NV293 in the inoculated samples. Of *Bet v1*-type PR (Pathogenesis-Related) genes not also annotated as “polyketide cyclase/dehydrase and lipid transport superfamily protein,” all of those differentially expressed between control plants and those inoculated with *B. fabae* were upregulated in response to inoculation (**Figure 4**). Enrichment analysis of GO terms showed that transcripts differentially expressed between NV293 and NV640 were enriched for terms related to multiple secondary metabolic functions, responses to oxidative stress, detoxification, signalling, and organelle membranes (**Supplementary Table S4**), but not for defence-related terms. An unknown transcription factor (DUF4371) was ranked second and was significantly upregulated in response to *B. fabae* in NV640, but not in NV293. Several leucine-rich repeat-containing protein kinases were among the differentially expressed genes, most of which were more strongly upregulated in NV640.

## 4 Discussion

A broad range of partial resistance to *B. fabae* was observed among the progeny of NV640 (resistant) and NV293 (susceptible) strains, which did not surpass the partial resistance observed in the resistant parent (**Supplementary Figure S2**, **Supplementary Table S9**). The paucity of positive transgressive segregants in the progeny also indicated that resistance was primarily inherited from the resistant parent NV640. The AUDPCs were normally distributed, suggesting that multiple genes were likely to be involved in this response, which is consistent with the identification of multiple QTLs with small effects in this study.

Unlike the gene-for-gene resistance reported for biotrophs and hemibiotrophs, resistance to necrotrophs has been found to be multigenic and often partial in nature, as reviewed by Corwin and Kliebenstein (2017), Liao et al. (2022), and Bi et al. (2023). In contrast to effector-triggered immunity (ETI) to biotrophs, conferred by major resistance genes, many small-effect QTLs have been found to be linked to partial resistance to necrotrophs, e.g. to *Botrytis cinerea* in *Arabidopsis thaliana* (Corwin et al., 2016), tomato (Finkers et al., 2007), and grape vine (Su et al., 2023); *A. fabae* (Ocaña-Moral et al., 2017) and *B. fabae* (Gela et al., 2022) in *V. faba*, and to *Mycosphaerella pinodes* in pea (Timmerman-Vaughan et al., 2002, 2004). In a large GWAS study in *A. thaliana* inoculated with multiple isolates of *B. cinerea*, a small number of receptor-like kinases and NLRs involved in PAMP-triggered immunity-type pathogen recognition and signalling were identified along with other biological functions, including transcription, RNA modification, signalling, vesicular transport, and detoxification among more than 3,000 candidate genes (Corwin et al., 2016). Similarly, many small-effect QTLs linked to the resistance of *V. faba* to *B. fabae* found in our study were enriched for functions and processes related to defence, as well as signalling, transport, vesicular and trans-membrane localisation, nucleic acid binding and transcription, tissue development, and oxidoreductase (**Supplementary Table S4**).

QTLs identified in the NV640 × NV293 population differed between years and stages of the disease, which may have been partly due to the different stages of the plants at inoculation, which was completed 57 days after planting (d.a.p.) in 2017, 95 d.a.p. in 2019, and 45 d.a.p. in 2021. For example, QTLs LG1@168.9, LG3@136.5, and LG3@144.5 were associated with the early stage of the disease in the field and contained several classes of genes that were significantly differentially expressed in seedlings shortly after inoculation between NV640 and NV293, e.g., *GNK2-*like proteins, associated with the recognition of mannose in fungal cell walls. QTLs detected at later stages were enriched for a diverse range of functions and processes, such as transport/localisation, tissue development, transcription, nucleic acid-binding, and oxidation–reduction (**Supplementary Table S4**), similar to many small QTLs detected in *A. thaliana* in response to *B. cinerea* (Corwin et al., 2016). Multiple QTLs on LGs 2, 3, and 6 were found to overlap with QTLs previously linked to Ascochyta leaf blight resistance in the *V. faba* cross 29H × Vf136 (Ocaña-Moral et al., 2017; Gutierrez and Torres, 2021) (**Supplementary Table S8A**). Additionally, all but one QTL on Linkage Group 3 were found to correspond to QTLs linked to resistance to *A. rabiei* from chromosome 4 in chickpea (2022), compiled by Alo et al. (2022) (**Supplementary Table S8B**). Although this could be coincidental, it could also be that the same or similar genes residing in QTL regions contribute to resistance against multiple different necrotrophs, as they contain clusters of genes linked to PAMP-triggered immunity (PTI), e.g., chitin receptors, receptor-like kinases, and genes annotated with salt stress response/antifungal functions (**Table 2**). Five QTLs have previously been mapped on *V. faba* chromosomes 1 and 6 for resistance to chocolate spot caused by *B. fabae* in an RIL population of cv. Mélodie/2 (S) × ILB 938/2 (R) (Gela et al., 2022). The QTLs LG6@110.0a and LG6@110.0b identified in this study overlapped with QTL qFB6.1; however, the other four QTL were not detected. This underlines the need to investigate multiple sources of partial resistance at multiple growth stages in well-replicated QTL studies and to align differential expression studies encompassing infection in seedlings and following the development of disease in mature plants.

All loci identified in linkage groups 2 to 6 confer resistance derived from NV640; however, a single QTL LG1@168.9, detected at the early stages of chocolate spot disease in 2021, confers resistance derived from NV293 (**Supplementary Figures S5**-**S7**). However, the difference in symptoms between the resistant and susceptible parents at that stage was only 2%, and QTL accounted for 22% of the variation.

To further elucidate the genes and pathways involved in early disease development, we conducted a differential expression analysis on NV640 and NV293 seedlings in response to the same combination of *B. fabae* isolates used in the field experiments. The time point represents the very early stage symptom development when lesions began to appear in both accessions, a time point that corresponded to the stage of disease observed in the first field assessments.

The most significantly differentially expressed disease resistance-like gene at the seedling stage was a wall-associated galacturonan-binding receptor kinase (WAK), similar to *Lr-10*. The *Lr-10* like wall associated kinase was also the most significantly differentially expressed gene in response to *B. fabae* in both parents compared to the uninoculated controls. It is upregulated in response to infection but to a much greater extent in NV293 cells. Overexpression of *Lr-10* has been found to increase resistance to brown rust in wheat, a biotrophic pathogen, by inducing a strong hypersensitivity response marked by chlorosis and localised tissue necrosis (Feuillet et al., 2003; Faris et al., 2010). This reaction, while effective against biotrophs, might be exploited by a necrotrophic pathogen such as *B. fabae*, thereby increasing the susceptibility of the host. NV293 and NV640 could, therefore be considered to differ in susceptibility to *B. fabae* rather than resistance. WAKs have previously been reported to contribute to resistance to *B. cinerea* in Rose (Liu et al., 2021) and *Magnaporthe oryzae* in rice; however, certain WAKs have also been demonstrated to increase susceptibility (Delteil et al., 2016). Therefore, it is possible that higher gene expression of specific WAKs could potentiate greater susceptibility to *B. fabae* in NV293 than in NV640.

Several other classes of defence-related genes, mostly associated with PTI against hemibiotrophs and necrotrophs, are differentially expressed in response to *B. fabae*. PAMP-triggered immunity, including the recognition of pathogen-associated molecular patterns (PAMPs) by WAKs, has been reported to be involved in regulating partial and broad-spectrum resistance to necrotrophs including *Botrytis* spp (Kohorn and Kohorn, 2012; Mengiste, 2012; Liao et al., 2022; Bi et al., 2023), and similar regulatory pathways could be responsible for the *V. faba* and *B. fabae* pathosystems. The genes that contribute to resistance or susceptibility in NV640 and NV293 remain unknown and would require a differential gene expression study over a time series overlapping with all stages of disease observed in the QTL study. Genes differentially expressed between NV640 and NV293 were also significantly enriched for GO terms related to various metabolic processes, reduction/oxidation, and membranes as cellular components. Previous studies have observed differential regulation of ROS-burst following inoculation with *B. fabae* between resistant and susceptible cultivars, which may contribute to partial resistance by limiting tissue damage and necrosis (El-Komy, 2014; Castillejo et al., 2021), indicating that similar regulatory mechanisms could contribute to resistance in NV640.

No complete sources of resistance against chocolate spots have been reported for faba bean, and it is likely that resistance to *B. fabae* is quantitative and partial rather than qualitative, similar to *B. cinerea* (Bi et al., 2023). Quantitative resistance to *B. fabae* is therefore likely to be polygenic, and improvement of resistance in bean varieties will require the selection of a combination of many small-effect loci, aided by the identification of multiple sources of resistance.

This supports the proposition that the identification of candidate genes responsible for differing resistance will require additional time-series expression studies, possibly involving multiple sources of partial resistance. This might elucidate which processes and QTLs are involved at different stages of disease progression, e.g., in the initiation phase of the infection or the transition to the aggressive stage. The mechanisms underlying partial resistance in NV640 and other partially resistant *V. faba* accessions remain unknown. Studies on collections of *V. faba* accessions for chocolate spot resistance in different countries and years have observed large genotype × environment interactions affecting the speed of disease progression and the ranking of lines, despite applying identical inoculation methods (Villegas-Fernández et al., 2009, 2012). This highlights the large environmental component of chocolate spot epidemics, which complicates the identification of reproducible quantitative QTLs, particularly under field conditions. It was also evident in this study that genes contributing to resistance to *B. fabae* might be overlooked in field-based QTL studies because of differences in plant development, assessment stage, and influences from environmental factors, especially under field conditions. Well-replicated assessments of seedlings and adult plants in controlled environments in addition to field experiments may be required to increase the chances of robust identification of small-effect QTL at different stages of the disease.

Despite the difficulties in elucidating the specific mechanism conferring partial resistance to chocolate spots, the introduction of multi-genic, quantitative resistance into elite varieties would still be valuable for limiting the heavy yield losses caused by the aggressive form of the disease, complicated by the loss of key active ingredients.

## Data availability statement

The datasets presented in this study can be found in online repositories. The names of the repository/repositories and accession number(s) can be found in the article/**Supplementary Material**.

## Author contributions

AW: Conceptualization, Data curation, Formal analysis, Funding acquisition, Investigation, Methodology, Supervision, Validation, Visualization, Writing – original draft, Writing – review & editing. TR: Data curation, Formal analysis, Investigation, Methodology, Writing – review & editing. TW: Data curation, Formal analysis, Investigation, Methodology, Validation, Visualization, Writing – review & editing. RC: Investigation, Methodology, Writing – review & editing, Supervision. DL: Supervision, Writing – review & editing, Project administration. JT: Project administration, Supervision, Writing – review & editing, Conceptualization, Funding acquisition, Resources, Validation. TW: Conceptualization, Funding acquisition, Project administration, Resources, Supervision, Validation, Writing – review & editing, Data curation, Formal analysis, Investigation, Methodology, Writing – original draft.

## Supplementary material

Supplementary Figure 1 Progression of chocolate spot symptoms in parents NV293 (S) and NV640 (R) plotted against rainfall, temperature, and relative humidity at weather station Cambridge-NIAB. Weather data displayed from planting to month of last scoring. **(A)** 2017, **(B)** 2019, **(C)** 2021.

Supplementary Figure 2 Correlation plot for areas under the disease progress curve (AUDPC) for chocolate spot leaf symptom coverage from field experiments in 2017 (F3 recombinant inbred lines, RILs), 2019 (F5 RILs) and 2021 (F6 RILs). Histograms for AUDPCs of the NV640 x NV293 progeny are also shown, where Y-axes: frequency of observation; AUDPC-scores for the parents are represented by arrows with NV640 (light blue, resistant) and NV293 (dark blue, susceptible). Asterisks corresponding to p-values of correlation: ***0, **0.001; *0.01.

Supplementary Figure 3 QTLs for AUDPCs for NV640 xNV293, F3, F5 and F6 families in 2017 **(A)**, 2019 **(B)** and 2021 **(C)** with alpha = 0.05 thresholds. Linkage group numbers correspond to chromosomes of *Vicia faba*.

Supplementary Figure 4 QTLs for chocolate spot scores in three field trials of NV640 x NV293 cross at 13, 15 and 19 days after inoculation (d.a.i.) for F3 in 2017 **(A-C)**, 3, 7, 10 and 13 d.a.i. for F5 in 2019 **(D-G)**, and 15, 32 and 44 d.a.i. for F6 in 2021 (panels H-J). Threshold at alpha = 0.05 (dashed line). Linkage group identifiers correspond to numbering of chromosomes of *Vicia faba*.

Supplementary Figure 5 Phenotype x Genotype plots for peak markers of QTLs detected in 2017. Peak marker names below plots. Genotypes: AA: parent NV640 (R), BB: parent NV293 (S), AB: heterozygous. Y-axes: foliar disease score at *n* days after inoculation (d.a.i.) or area under the disease curve (AUDPC).

Supplementary Figure 6 Phenotype x Genotype plots for peak markers of QTLs detected in 2019. Peak marker names below plots. Genotypes: AA: parent NV640 (R), BB: parent NV293 (S), AB: heterozygous. Y-axes: foliar disease score at *n* days after inoculation (d.a.i.) or area under the disease curve (AUDPC).

Supplementary Figure 7 Phenotype x Genotype plots for peak markers of QTLs detected in 2021. Peak marker names below plots. Genotypes: AA: parent NV640 (R), BB: parent NV293 (S), AB: heterozygous. Y-axes: foliar disease score at *n* days after inoculation (d.a.i.) or area under the disease curve (AUDPC).
