# Supplementary figures and images for: Identification of Faba bean genetic loci associated with quantitative resistance to the fungus Botrytis fabae, causal agent of chocolate spot
Source: Front Plant Sci. 2024 Apr 19;15:1383396. doi: 10.3389/fpls.2024.1383396 (PMC11067873; doi:10.3389/fpls.2024.1383396)

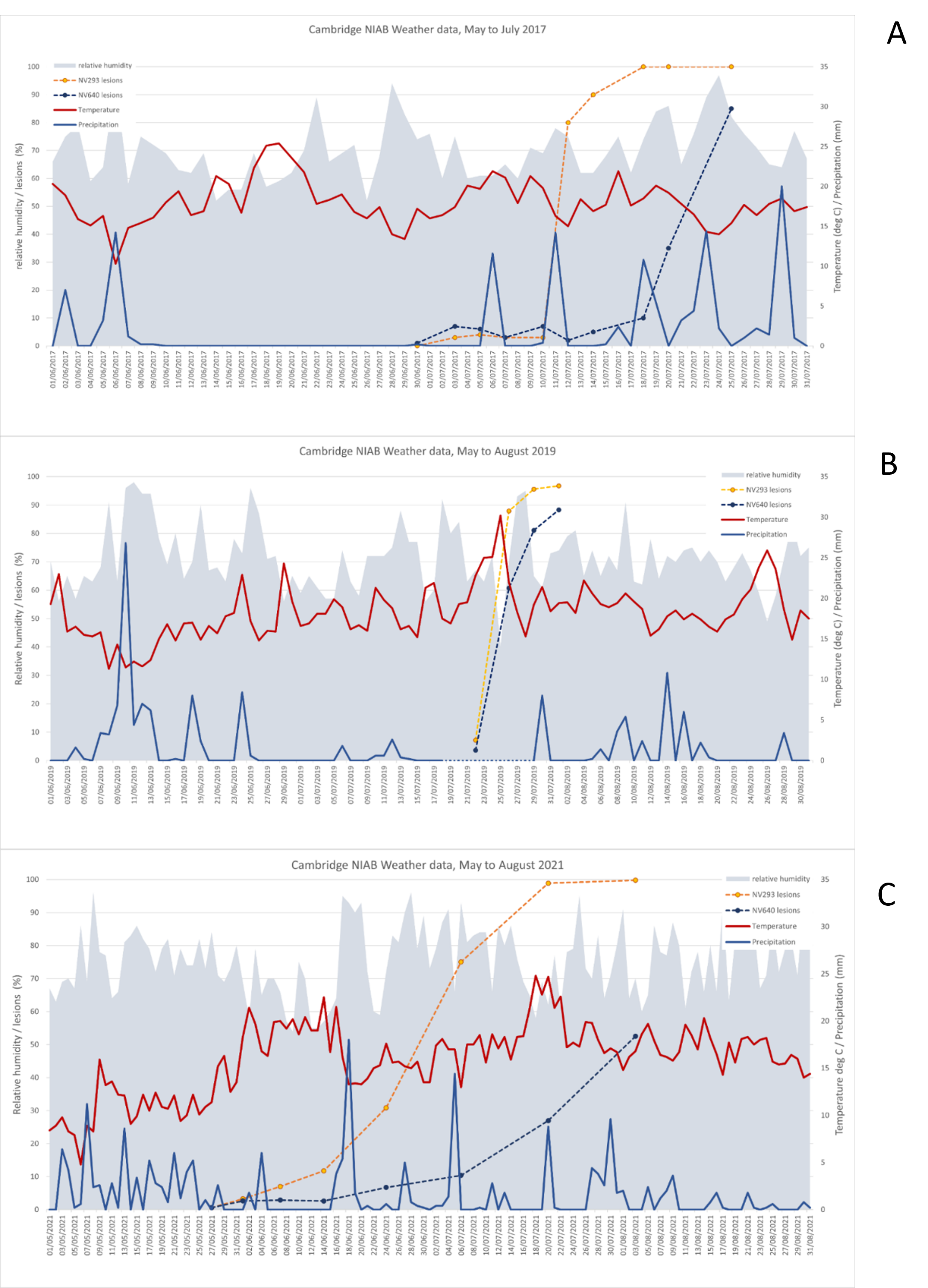

Supplement: Supplementary Figure 1 — Progression of chocolate spot symptoms in parents NV293 (S) and NV640 (R) plotted against rainfall, temperature, and relative humidity at weather station Cambridge-NIAB. Weather data displayed from planting to month of last scoring. (A) 2017, (B) 2019, (C) 2021. [file Image_1.tif]

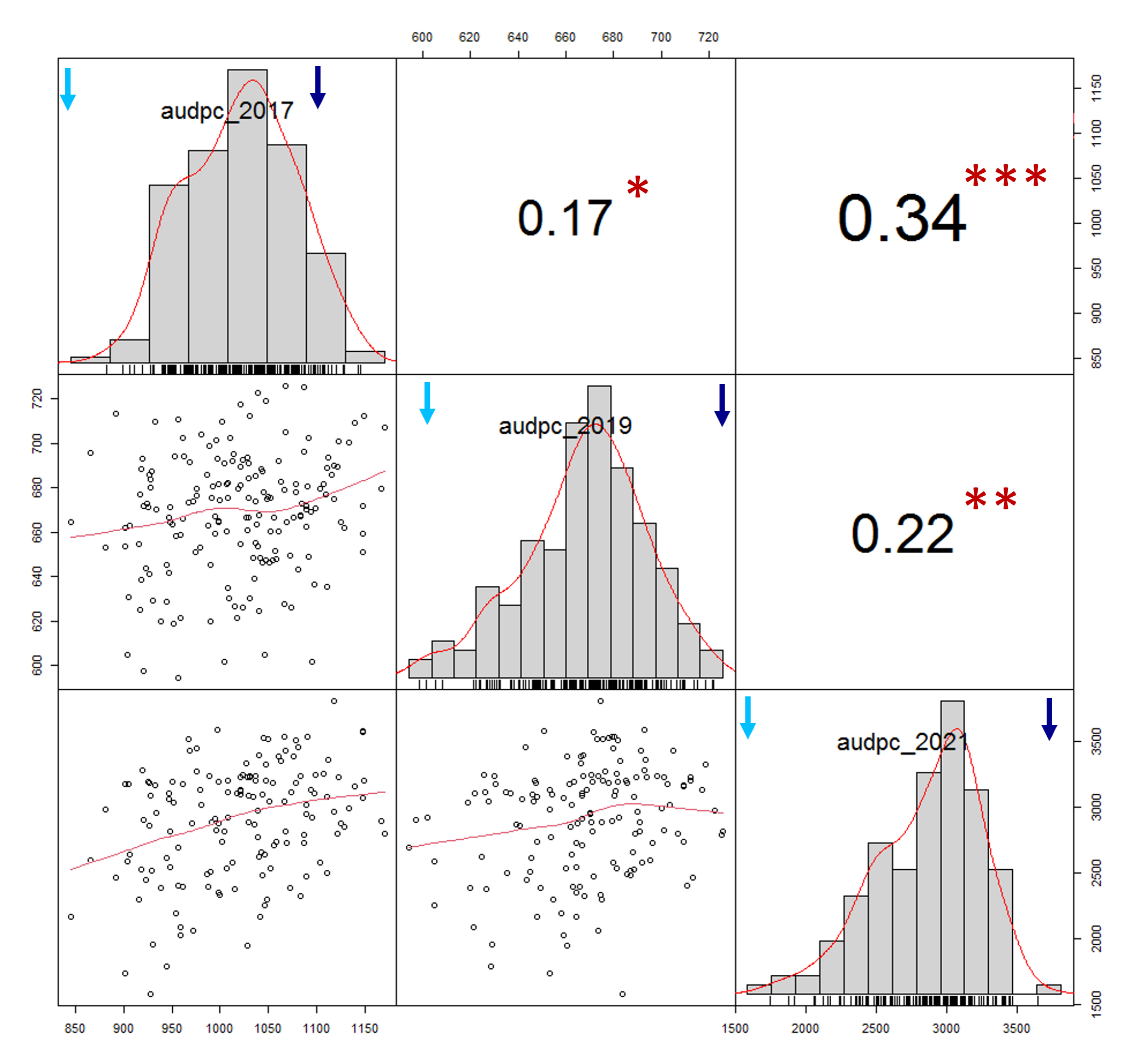

Supplement: Supplementary Figure 2 — Correlation plot for areas under the disease progress curve (AUDPC) for chocolate spot leaf symptom coverage from field experiments in 2017 (F3 recombinant inbred lines, RILs), 2019 (F5 RILs) and 2021 (F6 RILs). Histograms for AUDPCs of the NV640 x NV293 progeny are also shown, where Y-axes: frequency of observation; AUDPC-scores for the parents are represented by arrows with NV640 (light blue, resistant) and NV293 (dark blue, susceptible). Asterisks corresponding to p-values of correlation: ***0, **0.001; *0.01. [file Image_2.tif]

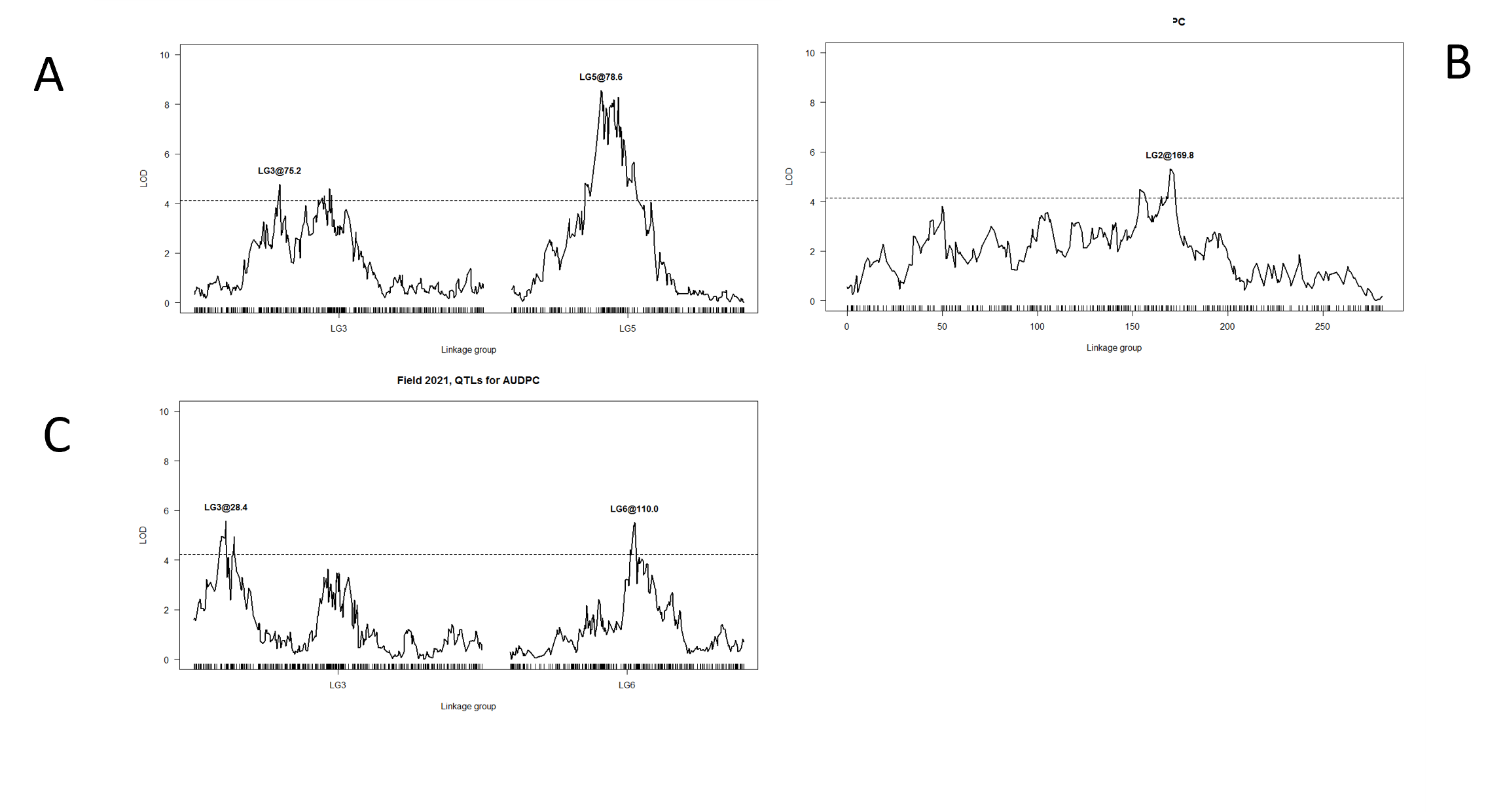

Supplement: Supplementary Figure 3 — QTLs for AUDPCs for NV640 xNV293, F3, F5 and F6 families in 2017 (A), 2019 (B) and 2021 (C) with alpha = 0.05 thresholds. Linkage group numbers correspond to chromosomes of Vicia faba. [file Image_3.tif]

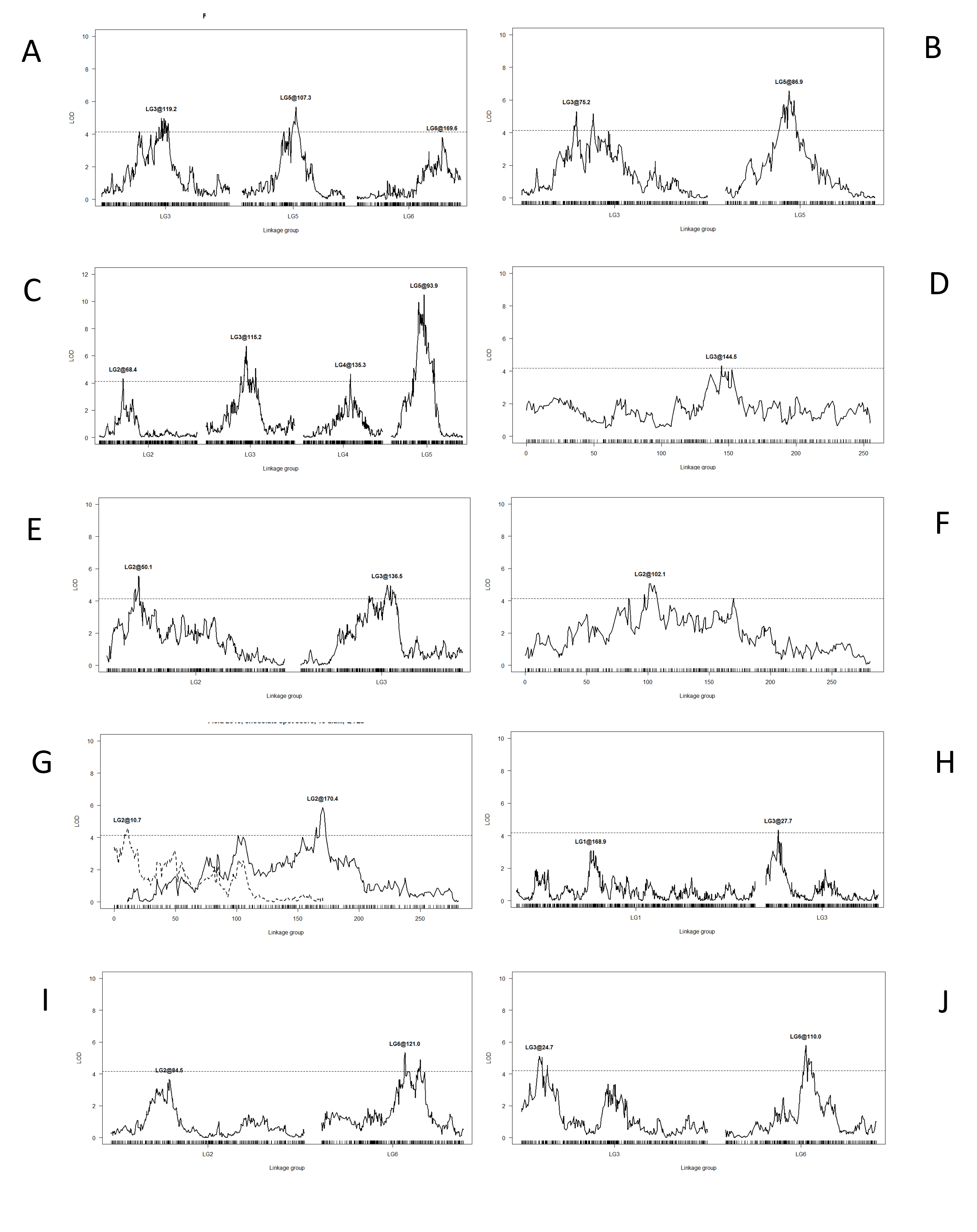

Supplement: Supplementary Figure 4 — QTLs for chocolate spot scores in three field trials of NV640 x NV293 cross at 13, 15 and 19 days after inoculation (d.a.i.) for F3 in 2017 (A-C), 3, 7, 10 and 13 d.a.i. for F5 in 2019 (D-G), and 15, 32 and 44 d.a.i. for F6 in 2021 (panels H-J). Threshold at alpha = 0.05 (dashed line). Linkage group identifiers correspond to numbering of chromosomes of Vicia faba. [file Image_4.tif]

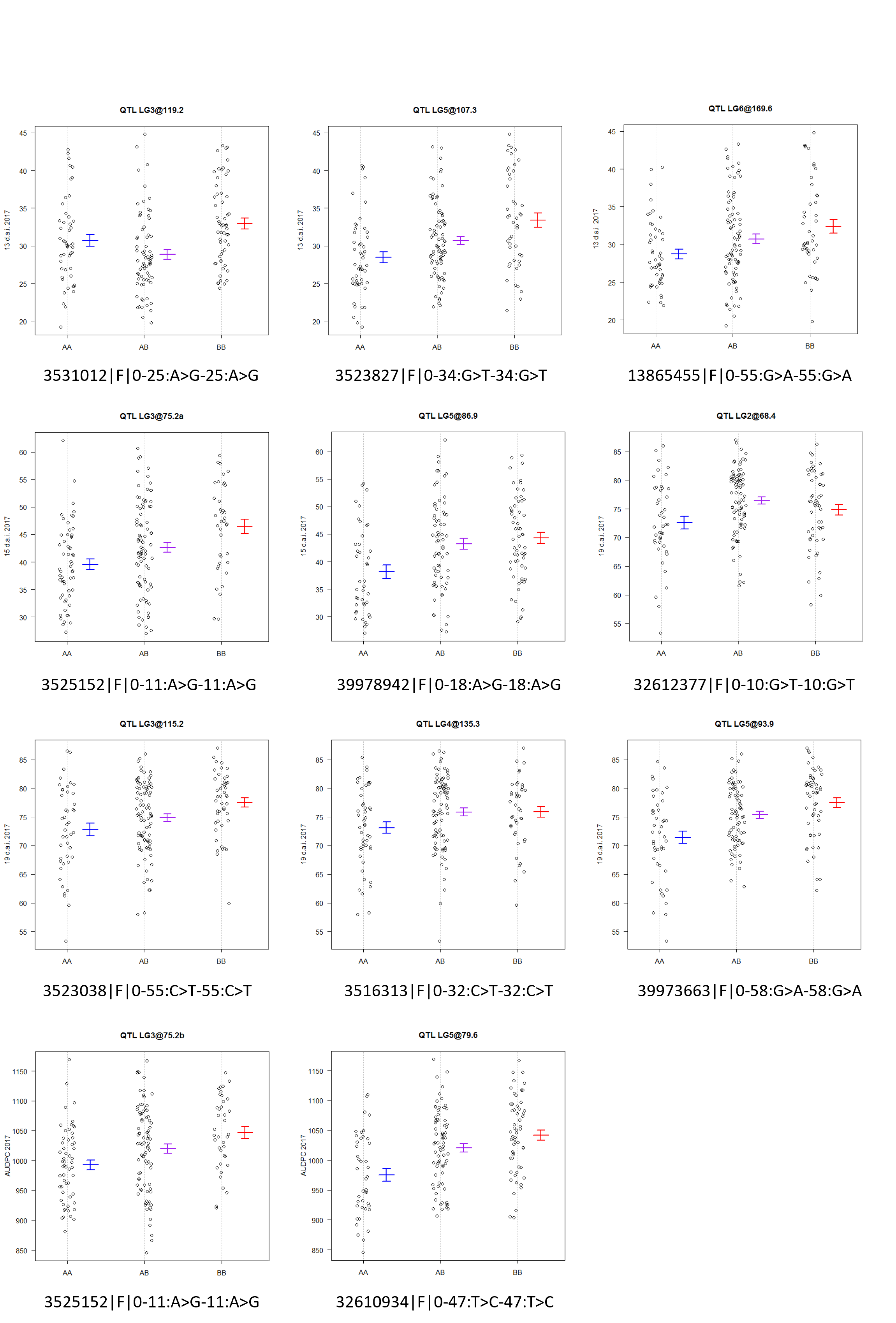

Supplement: Supplementary Figure 5 — Phenotype x Genotype plots for peak markers of QTLs detected in 2017. Peak marker names below plots. Genotypes: AA: parent NV640 (R), BB: parent NV293 (S), AB: heterozygous. Y-axes: foliar disease score at n days after inoculation (d.a.i.) or area under the disease curve (AUDPC). [file Image_5.tif]

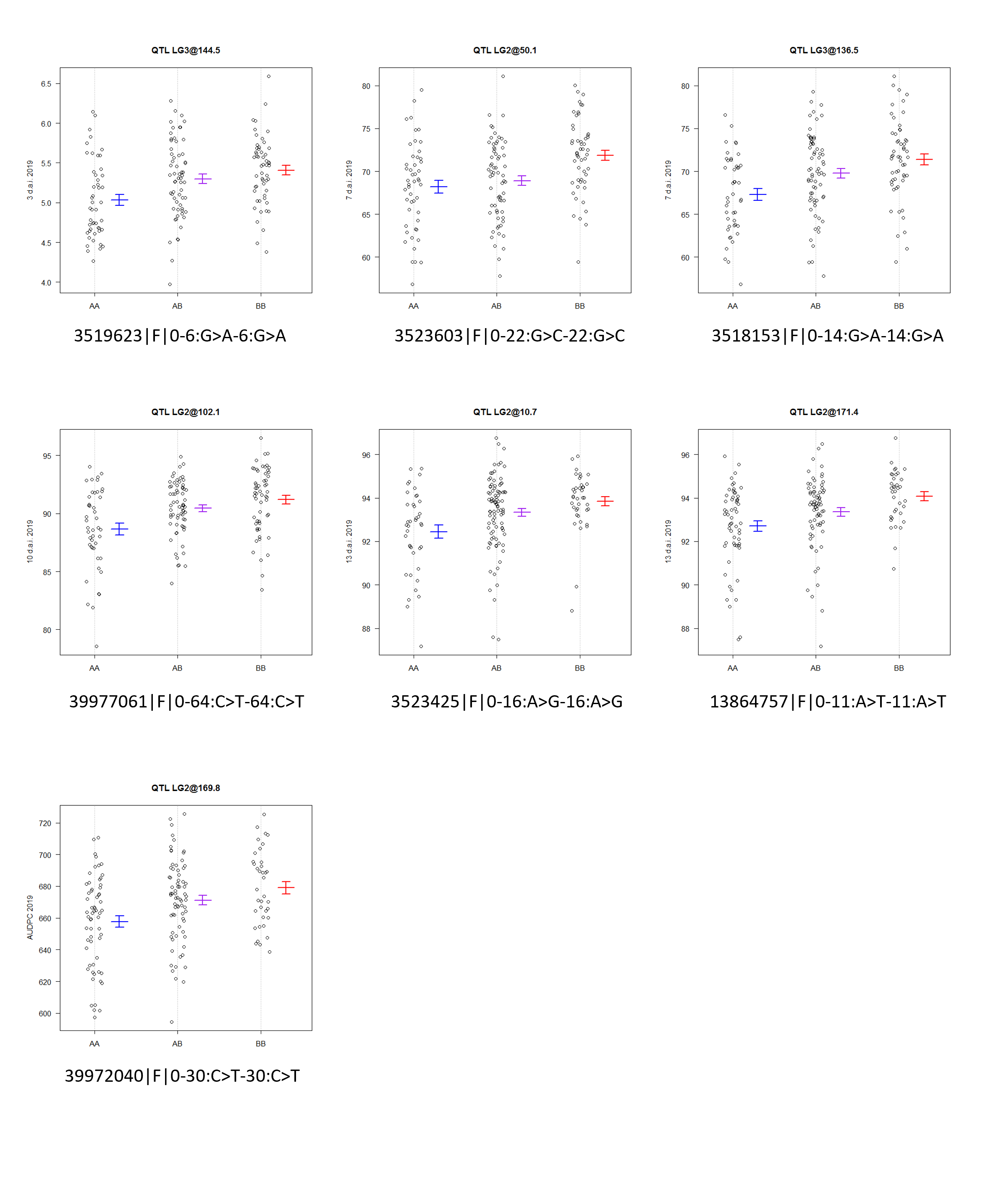

Supplement: Supplementary Figure 6 — Phenotype x Genotype plots for peak markers of QTLs detected in 2019. Peak marker names below plots. Genotypes: AA: parent NV640 (R), BB: parent NV293 (S), AB: heterozygous. Y-axes: foliar disease score at n days after inoculation (d.a.i.) or area under the disease curve (AUDPC). [file Image_6.tif]

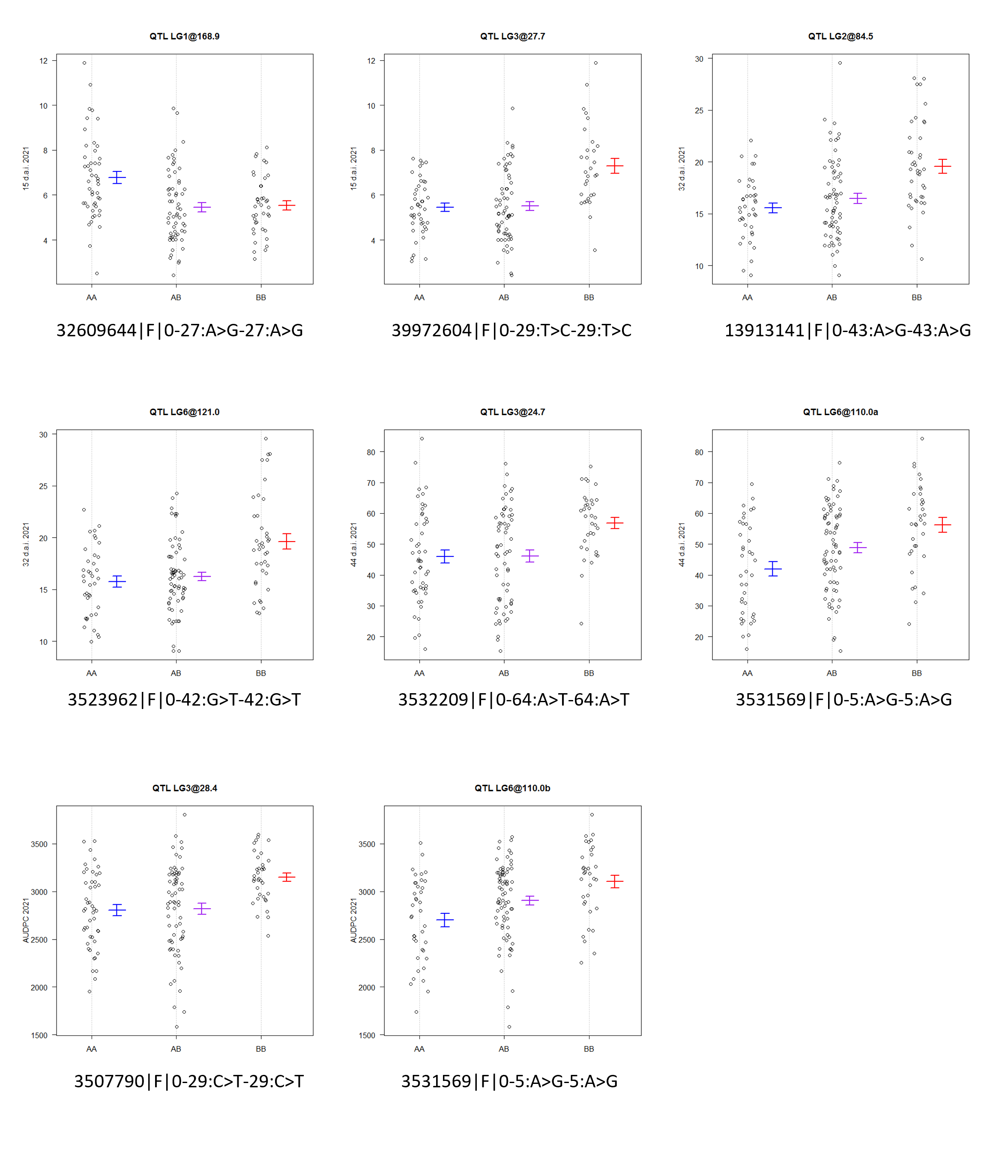

Supplement: Supplementary Figure 7 — Phenotype x Genotype plots for peak markers of QTLs detected in 2021. Peak marker names below plots. Genotypes: AA: parent NV640 (R), BB: parent NV293 (S), AB: heterozygous. Y-axes: foliar disease score at n days after inoculation (d.a.i.) or area under the disease curve (AUDPC). [file Image_7.tif]
